# Beyond blacklists: a critical assessment of exclusion set generation strategies and alternative approaches

**DOI:** 10.1093/bioinformatics/btag110

**Published:** 2026-03-13

**Authors:** Brydon P G Wall, Jonathan D Ogata, My Nguyen, Amy L Olex, Konstantinos V Floros, Anthony C Faber, Joseph L McClay, J Chuck Harrell, Mikhail G Dozmorov

**Affiliations:** Department of Biostatistics, Virginia Commonwealth University, Richmond, VA 23298, United States; Department of Biostatistics, Virginia Commonwealth University, Richmond, VA 23298, United States; Department of Biostatistics, Virginia Commonwealth University, Richmond, VA 23298, United States; C. Kenneth and Diane Wright Center for Clinical and Translational Research, Virginia Commonwealth University, Richmond, VA 23298, United States; VCU Philips Institute, Virginia Commonwealth University School of Dentistry and Massey Comprehensive Cancer Center, Richmond, VA 23298, United States; Department of Pediatrics, Virginia Commonwealth University, Richmond, VA 23298, United States; VCU Philips Institute, Virginia Commonwealth University School of Dentistry and Massey Comprehensive Cancer Center, Richmond, VA 23298, United States; Department of Pediatrics, Virginia Commonwealth University, Richmond, VA 23298, United States; Department of Pharmacotherapy and Outcomes Science, Virginia Commonwealth University, Richmond, VA 23298, United States; Department of Pathology, Virginia Commonwealth University, Richmond, VA 23284, United States; Massey Comprehensive Cancer Center, Virginia Commonwealth University, Richmond, VA 23298, United States; Department of Biostatistics, Virginia Commonwealth University, Richmond, VA 23298, United States; Department of Pathology, Virginia Commonwealth University, Richmond, VA 23284, United States

## Abstract

**Motivation:**

Short-read sequencing data can be affected by alignment artifacts in certain genomic regions. Removing reads overlapping these exclusion regions, previously known as Blacklists, help to potentially improve biological signal. Alternatively, “sponge” or decoy sequences have been proposed to reduce alignment artifacts.

**Results:**

We examined the widely used Blacklist software and found that pre-generated exclusion sets were difficult to reproduce due to sensitivity to input data, aligner choice, and read length. We further explored the use of “sponge” sequences—unassembled genomic regions such as satellite DNA, ribosomal DNA, and mitochondrial DNA—as an alternative approach. We additionally investigated the effect of the T2T-CHM13 genome assembly on improving biological signals. Aligning reads to a genome that includes sponge sequences reduced signal correlation in ChIP-seq data comparably to Blacklist-derived exclusion sets while preserving biological signal. Sponge-based alignment also had minimal impact on RNA-seq gene counts, suggesting broader applicability beyond chromatin profiling. These results highlight the limitations of fixed exclusion sets, and recommend the use of the T2T-CHM13 assembly or, for the hg38 genome assembly, “sponge” sequences as an alignment-guided strategy for reducing artifacts and improving functional genomics analyses.

## 1. Introduction

Alignment of short-read sequencing data to reference genome assemblies poses significant challenges due to the presence of low-complexity regions, centromeres, telomeres, satellite repeats, chromatin accessibility biases, and other artifacts (Miga et al. 2015). These regions often result in abnormal read pileups caused by ambiguous alignments or biases introduced during library preparation steps, such as cell fixation or PCR amplification after adapter ligation (Haile et al. 2019, Tanaka et al. 2020). Such artifacts have been observed across multiple species (Boyle et al. 2014, Wreczycka et al. 2019) and are particularly pronounced in chromatin-targeting sequencing technologies, including ChIP-seq (Kharchenko et al. 2008, Xie et al. 2013, Wreczycka et al. 2019), ChIP-exo (Carroll et al. 2014), and CUT&RUN (Nordin et al. 2022). Removing these artifact signals has been shown to reduce noise and improve peak calling, signal normalization, and motif analysis accuracy (Bailey et al. 2013, Hoffman et al. 2013, Carroll et al. 2014, Tanaka et al. 2020, Nordin et al. 2022). Hence, defining strategies for removing such problematic signals is crucial for accurate genomic data analysis and interpretation.

Lists of exclusion regions (formerly known as blacklist regions), defined as genomic coordinates of problematic regions, have become a commonly accepted method for excluding artifact signals (Ogata et al. 2023). Several exclusion region sets have been developed for humans and model organisms, with an overview provided by (Carroll et al. 2014). The ENCODE project and others have compiled exclusion sets for various genome assemblies (Amemiya et al. 2019, Wimberley and Heber 2020, Klasfeld and Wagner 2022). The GRCh38/hg38 human genome assembly remains indispensable due to its extensive history in genomic literature, deep integration into clinical databases, and wealth of existing functional annotations, underscoring the need for standardized exclusion set definitions and reproducible methods for generating them.

Methods for creating exclusion sets range from ad hoc approaches (Scheinin et al. 2014, Wreczycka et al. 2019, Nordin et al. 2022) to dedicated tools. Among these, the Blacklist software (Amemiya et al. 2019) is widely used. This tool employs BAM files from ChIP-seq input experiments (expected to have uniform non-specific sequencing coverage) and mappability files generated by Umap (Karimzadeh et al. 2018). However, several key parameters of its algorithmic implementation are not fully documented, including the number of BAM files required, the read length and aligner specifications, and the k-mer length for selecting low-mappability files. The impact of these parameters on exclusion set generation remains unclear. Additionally, the software merges regions within a fixed distance of 20 000 bp—a parameter that appears excessive and is not easily adjustable. While these simplifications allow for straightforward use as a black-box tool, they raise concerns about the robustness and biological relevance of the resulting regions, limiting its applicability across sequencing technologies and reference genomes.

Alternative methods also have limitations. PeakPass employs a random forest model trained on hg19 ENCODE blacklist regions to predict excludable regions in hg38. It identifies assembly gaps and genome complexity as key predictors but lacks a user-friendly implementation. Similarly, Greenscreen (Klasfeld et al. 2022) uses a traditional approach by processing ChIP-seq data through standard pipelines and calling peaks with MACS2 (Zhang et al. 2008). While Greenscreen claims 99% agreement with Blacklist-generated regions, its reliance on fixed parameters and a technically challenging Docker-based setup hampers its utility. The GreyListChIP R package (Brown 2015), developed in 2015, defines excludable regions using a tiled genome and merges regions within a fixed distance. However, its unpublished algorithm and untested parameters further limit its reliability, particularly for less-studied organisms.

An alternative approach involves aligning sequencing data to reference genomes that include so-called “decoy” or “sponge” sequences, which encompass unmapped and uncharacterized regions, satellite repeats, ribosomal sequences, and mitochondrial sequences. Incorporating these sponge sequences into the genome assembly during alignment has been shown to reduce signal in Blacklist exclusion regions and mitigate other alignment artifacts (Miga et al. 2015). Despite its advantages, this approach is less widely adopted, with only 49 citations compared to 1914 citations for the Blacklist manuscript (Google Scholar, February 2026).

The Telomere-to-Telomere (T2T) CHM13 human genome assembly offers a comprehensive alternative strategy for mitigating alignment artifacts. Because T2T-CHM13 resolves complex regions such as centromeres, telomeres, and segmental duplications, it theoretically contains the endogenous “sponge” sequences that were missing from previous assemblies (Nurk et al. 2022). Aligning directly to this gapless reference may reduce ambiguous mapping and artifactual pileups by providing accurate genomic targets for reads originating from these difficult repetitive regions.

In this study, we systematically benchmarked the performance of the Blacklist software (Amemiya et al. 2019) and related factors, such as aligner choice, to develop recommendations for exclusion set generation. Focusing on human and mouse genomes, we identified reproducibility and quality issues in existing exclusion sets. We provide a corrected, configurable version of the Blacklist software and propose improvements for defining exclusion regions. Rather than relying on fixed lists of exclusion regions, our results emphasize the importance of using “sponge” sequences at the alignment step. We also demonstrate that the T2T-CHM13 alignment strategy achieves comparable reductions in artifact signals while improving biological relevance. Our findings underscore the importance of transparent algorithms and adaptable methodologies to address the ongoing challenges in sequencing data analysis.

## 2. Methods

### 2.1. Data sources

The original Blacklist exclusion lists for human, mouse, and other species were obtained from the Blacklist GitHub repository (https://github.com/Boyle-Lab/Blacklist/tree/master/lists), using version 2 of the lists. The Kundaje Unified list was retrieved from the ENCODE Project (accession number ENCFF356LFX). The Nordin CUT&RUN exclusion set was originally obtained from Additional File 2 of the corresponding publication (Nordin et al. 2023) and sourced from the excluderanges R package (Ogata et al. 2023). Unless otherwise indicated, we used lists based on the hg38 human genome assembly.

To replicate the Blacklist exclusion lists, accession numbers of the corresponding BAM files were obtained using metadata provided in the Blacklist GitHub repository (https://github.com/Boyle-Lab/Blacklist/tree/master/lists/metadata) and downloaded from ENCODE. The “Umap” mappability files were obtained from the Hoffman Lab Umap/Bismap Project page (https://hoffmanlab.org/proj/bismap/). Accession numbers of the FASTQ files used to generate these BAM files were identified via the (**PG?**) tag in the header lines of each BAM file and downloaded from ENCODE.

The Gm12878 transcription factor FASTQ files used for correlation analysis were retrieved from ENCODE using the following metadata link: https://www.encodeproject.org/metadata/? type=Experiment&cart=%2Fcarts%2F4b50a1ed-d002-4c76-8401-5ac42d3f2228%2F&files.output_category=raw+data.

Experimental RNA-seq data (Boyd et al. 2023, Zboril et al. 2023) were obtained from GEO accession GSE235167. Experimental ATAC-seq data (Floros et al. 2025) were obtained from GSE266134.

The whole-genome sequencing (WGS) FASTQ files (ERR194147 for individual NA12878) were downloaded from https://www.ebi.ac.uk/ena/browser/view/ERR194147. The GIAB benchmark SNP/InDel dataset (HG001_GRCh38_1_22_v4.2.1) was downloaded from https://ftp-trace.ncbi.nlm.nih.gov/ReferenceSamples/giab/release/NA12878_HG001/latest/GRCh38/. For hg38 base recalibration, dbSNP build 155 was used in addition to known indels from UCSC (https://storage.googleapis.com/genomics-public-data/resources/broad/hg38/v0/Homo_sapiens_assembly38.known_indels.vcf.gz). For CHM13v2.0, we used the same dbSNP build 155 lifted over by the T2T consortium (https://s3-us-west-2.amazonaws.com/human-pangenomics/T2T/CHM13/assemblies/annotation/liftover/dbSNP.htm) to the appropriate coordinates. All data were accessed on August 12, 2025.

### 2.2. Generated exclusion sets

Blacklist exclusion sets for the hg38 and mm10 genome assemblies were generated using the BAM files listed in the metadata on GitHub, relevant Umap mappability files, and the original Blacklist software.

To define High Signal regions for the hg38 genome assembly, we generated a signal track by merging 274 paired-end 100/101 bp BAM files (aligned with bwa 0.7.10 sampe) via samtools merge (v1.2). We called local peaks using: macs3 callpeak—treatment ${in_file} —name 101_local—outdir ${out_dir} —gsize hs—slocal 10000—llocal 100000—keep-dup all (v3.0.1) and selected those with a fold change greater than 99.0% of the fold change range. Regions within 1000 bp were merged, and regions smaller than 1000 bp were discarded. This strict filtering ensured the selection of highly significant and long high-signal regions. After generating our High Signal regions, we merged these regions with centromeres from the UCSC Genome Browser, available via the excluderanges R package (Ogata et al. 2023).

To define Low Mappability regions for the hg38 genome assembly, we downloaded the kmer-100 multi-read mappability bedGraph file from the Hoffman Lab website (https://bismap.hoffmanlab.org), which contains base-level mappability values ranging from 0 to 1 (with 1 being highly mappable). We first defined the mappable universe by selecting regions with mappability greater than 0.01. Using bedtools (v2.31.1), regions within 1000 bp were merged. Inverting the mappable universe resulted in a set of regions with mappability ≤0.01. These regions were then merged within 1000 bp, and regions smaller than 1000 bp were discarded. This strategy preserved mappable regions interspersed with short unmappable segments while conservatively selecting long low-mappability regions.

The GreyListChIP R package generates a BAM file-specific exclusion set. We generated GreyListChIP lists for humans by combining 38 136 bp single-end FASTQ files used in the original Blacklist publication, realigning them with STAR to the hg38 genome assembly, and setting the maxGap parameter to 1,000. This list was used in our benchmarks. Additionally, we combined 274 paired-end 100 bp and 101 bp FASTQ files and generated lists for the hg38 and T2T genome assemblies. Similarly, for mice, we used 10 636 bp single-end FASTQ files and 17 650 bp single-end FASTQ files to generate lists for the mm10 and mm39 genome assemblies. All GreyListChIP lists have been added to our excluderanges R package (Ogata et al. 2023).

### 2.3. Comparison of exclusion sets

We compare two exclusion sets $A=\{a_{1},a_{2},\ldots ,a_{i}\}\;\text{and}\;B=\{b_{1},b_{2},\ldots ,b_{j}\}$ using the Jaccard count overlap, which is defined as the ratio between the average number of regions in A and B that overlap with any region in the other set, and the total number of unique regions in the union of A and B. In mathematical terms, this is given by


$$J_{c}(A,B)=\frac{\frac{1}{2}(\left|\left\{a\in A:a\cap B\neq \emptyset \right\}|+|\left\{b\in B:b\cap A\neq \emptyset \right\}\right|)}{\left|A\cup B\right|}.$$

Similarly, Forbes width overlap formula:


$$F_{w}(A,B)=\frac{\text{Genome}\;\text{Size}\cdot W(A\cap B)}{W(A)\cdot W(B)}$$

Where W(A) and W(B) are the total widths in bases of sets A and B, respectively, W(A∩B) is the total width of the intersection of A and B, and the “Genome Size” is the total reference genome size (e.g. 2 875 001 522 base pairs for hg38).

Similarity among exclusion sets was visualized using Classical Multidimensional Scaling (MDS) by converting the Jaccard count overlap and Forbes width overlap matrices into distance matrices and plotting exclusion sets using the first two principal coordinates. These distance matrices were also used for hierarchical clustering with the Ward clustering method. All other comparisons (e.g. number and width of overlapping regions) were performed using the GenomicRanges (v1.56.1) R package (Lawrence et al. 2013), R (v4.4.0), and Bioconductor (v3.19) (Gentleman et al. 2004).

### 2.4. Aligner test

The original hg38 BAM files were aligned using version 0.7.10 of the bwa sampe (paired-end) and bwa samse (single-end) aligners (Li and Durbin 2009). In our comparison of exclusion sets generated from BAMs aligned with different aligners, we used the following: bwa-mem2 2.2.1 (Li and Durbin 2009): bwa-mem2 mem references/GCA_000001405.15_GRCh38_no_alt_analysis_set.fna.gz <R1.fastq.gz> <R2.fastq.gz> | samtools view -bS—| samtools sort -o <bwamem.bam> -; bowtie2 2.5.4 (Langmead and Salzberg 2012): bowtie2 -x references/GCA_000001405.15_GRCh38_no_alt_analysis_set -U <R1.fastq.gz> <R2.fastq.gz> —local | samtools view -bS—| samtools sort -o <bowtie2.bam> -; STAR 2.7.11b (Dobin et al. 2013): STAR—genomeDir <references/star> —readFilesIn <(bgzip -cd <R1.fastq.gz>) <(bgzip -cd <R2.fastq.gz>) —outSAMtype BAM SortedByCoordinate—alignEndsType Local, as well as htslib and samtools 1.20 (Danecek et al. 2021).

### 2.5. Correlation analysis

FASTQ files for each transcription factor were merged, and the resulting files were aligned using STAR 2.7.11b (–alignEndsType Local) to GRCh38_no_alt_analysis_set_GCA_000001405.15.fasta (ENCODE ID: ENCSR425FOI) and to the same reference concatenated with “sponge” sequences (Miga et al. 2015).

Read count bins for each exclusion set or sponge were computed using deepTools v3.5.6 (Ramirez et al. 2014) with the multiBamSummary bins command (default settings). The Pearson correlation matrix was then obtained using plotCorrelation (–corMethod pearson), and each matrix was clustered and visualized in R using Euclidean distance and complete linkage.

### 2.6. RNA-seq analysis

We realigned our RNA-seq data (Boyd et al. 2023, Zboril et al. 2023) (GSE235167, *n* = 144) to two versions of the hg38 human genome assembly from the UCSC Genome Browser. One version included only autosomal, sex, and mitochondrial chromosomes (referred to as “autosomal”), while the other also contained additional random and unplaced contigs, as well as “sponge” sequences (Miga et al. 2015) (referred to as “full plus sponge”). Additionally, we utilized the T2T-CHM13v2.0 assembly (Nurk et al. 2022) and the corresponding UCSC GENCODEv35 CAT/Liftoff v2 gene annotations downloaded from https://github.com/marbl/CHM13. The data were processed using the Nextflow rnaseq v3.19.0 pipeline (Ewels et al. 2019) with the STAR-RSEM quantification strategy.

### 2.7. ATAC-seq analysis

The ATAC-seq data from synovial sarcoma SYO-1 cell line (GSE266134) were processed using the nf-core/atacseq v.2.1.2. We used the same genome assemblies as for RNA-seq (hg38 autosomal, hg38 full plus sponge, T2T). We defined peaks differentially accessible under treatment with the small molecule SAE1/2 inhibitor, TAK-981 (subasumstat) vs. untreated cells using macs2 v.2.2.9.1 with default settings for the human genome organism. Peaks were annotated with the annotatePeak function from the ChIPseeker v.1.44.0 R package (Yu et al. 2015), KEGG pathway enrichment analysis was performed with the enrichKEGG function from the clusterProfiler v.4.16.0 R package (Yu et al. 2012).

### 2.8. WGS analysis

Whole-genome sequencing (WGS) data were processed using the nf-core/sarek v3.5.0 pipeline with the BWA-MEM2 setting. We used the same genome assemblies as for RNA-seq (hg38 autosomal, hg38 full plus sponge, T2T). Variant calling was performed using GATK HaplotypeCaller within the nf-core pipeline. Variants (including the GIAB benchmark set) were normalized using BCFtools v1.19 (Danecek et al. 2021), removing duplicates and standardizing variant representation. Variants were annotated with dbSNP 155, and split by type (SNPs or INDELs) using BCFtools. T2T unique regions in comparison to hg38 obtained from the UCSC genome browser (“hub_3671779_hgUniqueHg38” table) were used to analyze variants in regions common to the hg38 genome assembly. Overlapping dbSNP ids were then used to analyze overlapping variants across assemblies.

### 2.9. Functional enrichment analysis

Hypergeometric enrichment analysis of transcripts with a non-zero sum of exon widths covered by exclusion regions was performed using the enrichR 3.2 R package (Kuleshov et al. 2016) and the “KEGG_2019_Human” signature database. Analysis of cancer drivers affected by exclusion sets was conducted using the oncoEnrichR 1.5.2 R package (Nakken et al. 2023), considering the “Cancer associations” analysis.

### 2.10. Excluderanges R package update

We added the GreyListChIP-generated sets to our excluderanges R package (Ogata et al. 2023), making them available within the R ecosystem. We used STAR-aligned sequencing data with 36 bp and 101 bp read lengths for the human genome and 36 bp and 50 bp read lengths for the mouse genome, applying a maxGap = 1,000 merge setting. Specifically, lists for the hg38 and T2T human genome assemblies and the mm10 and mm39 mouse genome assemblies were added.

## 3. Results

Filtering signal overlapping excludable (also known as Blacklist) regions is a standard practice in genomic data analysis. These regions were originally defined using input ChIP-seq data (ENCODE Project Consortium, 2012, Carroll et al. 2014, Amemiya et al. 2019). The simplicity of excludable regions (genomic coordinates in BED format) has enabled their application in analyzing genomic data generated by technologies based on assumptions other than ChIP-seq, such as ATAC-seq (Yan et al. 2020, Yang et al. 2020), chromatin conformation capture technologies (Huang et al. 2022), and their single-cell variants (Vitak et al. 2017, Chen et al. 2019, Heumos et al. 2023), as well as in pipelines and tools for genomic data analysis (Ramirez et al. 2014, Stansfield and Dozmorov 2017, Crowley et al. 2021).

The ENCODE Blacklist exclusion set is arguably one of the most cited and frequently used exclusion sets for the human genome, and the Blacklist software implementation has been employed to generate exclusion sets for model organisms (Amemiya et al. 2019). However, we have observed instances where signals expected to be associated with functionally relevant genes were missed, and such genes often overlapped with or were located in proximity to Blacklist exclusion regions. These observations prompted us to investigate the properties of the ENCODE Blacklist exclusion sets and compare their biological impact with alternative strategies, such as alignment to the hg38 human genome with the “sponge” sequences or the T2T-CHM13 genome assembly.

### 3.1. Challenges in reproducing pre-generated blacklist exclusion sets

The Blacklist GitHub repository offers pre-generated exclusion sets (also referred to as lists) for human (hg19, hg38), mouse (mm10), Drosophila (dm3, dm6), and worm (ce10, ce11). We noted that version 1 of the hg38 exclusion set contained 38 regions, in contrast to 636 regions in version 2. In an effort to reproduce the hg38 “GitHub Blacklist,” we ran the software using the same set of 250 BAM files described in the original publication, generating what we refer to as the “Generated Blacklist.” These experiments generated exclusion regions that differed from those available on GitHub. The “Generated Blacklist” contained more regions (1273 vs. 636 in the GitHub version, Table 1), which were narrower (mean width of approximately 213 Kbp vs. 357 Kbp in the GitHub version). We observed substantial differences in telomere/centromere/short arm overlaps, and these results were confirmed using the mouse mm10 Blacklist (Supplementary Results). To support reproducibility, we have made our analysis scripts available (Code Availability). These findings highlight variability in exclusion sets generated by the Blacklist software and suggest the value of cross-comparison with curated references like the “Kundaje Unified” list.

To better understand the differences between the exclusion sets provided on GitHub and those we generated, we examined the properties of the 1255 BAM files used to create the hg38 “GitHub Blacklist.” We observed a wide variety of characteristics among the input BAM files, from single/paired-end differences and read length differences (36–101 bp), sequencing depth, to differences in sample origin (Supplementary Results). We further hypothesized that aligner selection and read length could influence the resulting exclusion sets. To assess this effect, we used STAR, bwa-mem2, and bowtie2 aligners (Methods), along with the originally used bwa samse, to realign 38 136 bp single-end and 27 4101 bp paired-end files and generate exclusion sets using the Blacklist software. We observed substantial differences in exclusion set properties (Supplementary Results). These results suggest that the diversity in input data characteristics, along with differences in sequencing and alignment protocols, may influence the reproducibility and consistency of exclusion sets generated by the Blacklist software.

**Table 1:** Characteristics of the hg38 exclusion sets.

List	Total		Summary of region widths, bp			Number of regions/Coverage proportion of		
	Count	Coverage, bp	Minimum	Mean/Median	Max	Centromeres (%)	Telomeres (%)	Short arms (%)
GitHub Blacklist	636	227162400	1200	357174/10150	30590100	30/97.6	35/72.7	3/58.9
hg38 Generated List	1273	271267100	1000	213093/6300	30602600	42/96.7	32/66.5	4/83.7
mm10 GitHub List	3435	238977200	1000	69571/8100	50585400	72/2.7	17/35.4	86/23.4
mm10 Generated List	2970	253654600	1000	85406/12600	91744600	74/4.0	17/37.5	76/24.0
hg38 Kundaje Unified	910	71570285	19	78649/384	5407756	27/97.7	1/0.0	0/0.0
High Signal + Centromere	2831	71535766	1000	25269/3083	5400309	24/100.0	1/0.0	2/0.0
Low Mappability	3691	153220769	1001	41512/1823	18171354	984/28.7	46/95.8	5/100.0
HS + LM + CM	5409	206519467	1000	38181/2885	18223524	23/99.6	47/95.8	5/100.0
hg38 Nordin CUT&RUN	884	4463850	2	5050/2878	93434	555/4.2	7/1.4	0/0.0
GreyListChIP STAR 1k	22480	9270576	1	412/36	140768	5673/4.5	0/0.0	0/0.0

Total number of regions, average region width, number of regions overlapping gaps, and proportion of gap coverage.

### 3.2. Manually defined high signal and low mappability regions are different from other exclusion sets

**Figure 1:**
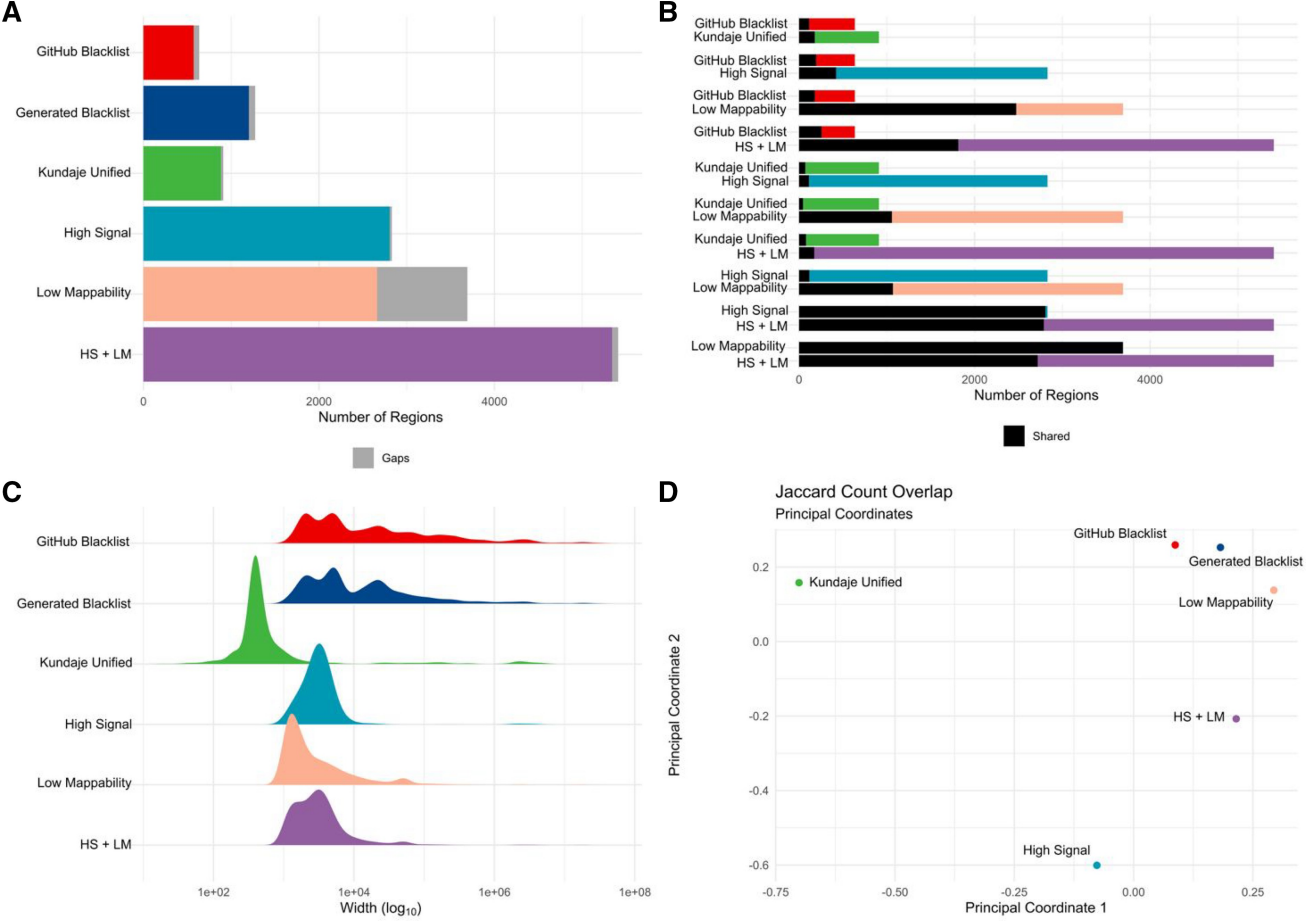
Differences between the GitHub version of the hg38 exclusion set and manually defined gold-standard exclusion sets. (A) Differences in count. (B) Pairwise overlaps of region counts between the GitHub exclusion set and manually defined gold-standard exclusion sets. (C) Differences in width distribution. (D) Multidimensional scaling plot of Jaccard count similarity among the GitHub exclusion set and manually defined gold standards.

To evaluate the reproducibility of exclusion sets in the absence of a definitive gold standard for excludable regions, we chose the manually curated “Kundaje Unified” list as our recommended reference for known hg38 exclusion regions (Ogata et al. 2023). This list includes a larger number of regions compared to the GitHub-provided exclusion set (910 regions in the “Kundaje Unified” list vs. 636 in the GitHub version, Fig. 1A). Of these, 179 regions overlapped with the GitHub list (Fig. 1B). The “Kundaje Unified” regions were generally narrower (mean width of 78.6 Kbp compared to 357.2 Kbp in the GitHub list, Fig. 1C) and covered less of the genome (71.6 Mbp for the “Kundaje Unified” list vs. 227.2 Mbp for the GitHub list, Table 1). In contrast to the GitHub list, the “Kundaje Unified” list did not cover telomeres and short arms (Supplementary Results). For our benchmarking, we used the “Kundaje Unified” list as the primary reference, recognizing its role as a curated and widely referenced set of exclusion regions.

High Signal (HS) regions are another well-characterized category of exclusion regions (Yang et al. 2020). Using 27 4101 bp paired-end BAM files, we identified “High Signal” regions by applying MACS3 to call peaks with fold changes exceeding the 99th percentile. We merged regions within 1000 bp and excluded those smaller than 1000 bp (Methods). Our analysis showed that the “High Signal” regions had limited coverage of centromeres compared to the “Kundaje Unified” list (2.1% vs. 97.7%). To address this, we incorporated centromeric regions into our “High Signal” list, producing a final set of 2657 regions (Fig. 1A). The resulting list differed from the “Kundaje Unified” and the GitHub Blacklist in width and overlap (Supplementary Results). This “High Signal” list offers a conservative representation of high-signal regions derived from input ChIP-seq data.

**Figure 2:**
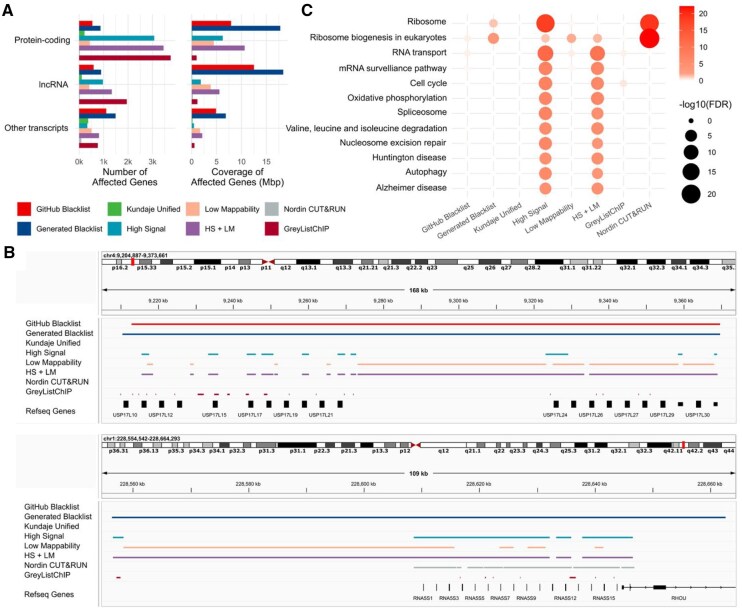
Biological characterization of genes affected by exclusion regions. (A) Count and coverage of protein-coding, long noncoding, and other transcripts affected by exclusion sets; (B) Representative comparison of exclusion set coverage over a cluster of ubiquitin-specific peptidase 17-like family member genes (top) and a cluster of ribosomal genes (bottom); (C) KEGG pathways enriched in genes overlapped by exclusion sets.

Low Mappability (LM) regions represent another category considered by the Blacklist software. Following an approach similar to that used for “High Signal” regions, we defined “Low Mappability” regions as those falling below the 1st percentile of the mappability range. Despite applying the same merging and filtering strategy as for the “High Signal” regions, we identified a substantial number of “Low Mappability” regions (12 455, Fig. 1A), which tended to be wide (mean width of 41.5 Kbp, Fig. 2B). Similar to the “High Signal” list, the “Low Mappability” regions differed from the “Kundaje Unified” and the GitHub Blacklist (Supplementary Results). This “Low Mappability” list reflects a conservative set of regions that are challenging to map.

To align with the characteristics of the Blacklist-generated list, which includes both high signal and low mappability regions, we combined the “High Signal” (with centromeres) and “Low Mappability” lists, referred to as “HS + LM”. This resulted in 5,409 regions with a mean width of 38.1 Kbp, covering 206.5 Mbp of the genome and nearly complete gap coverage (Fig. 1, Supplementary Results). The “HS + LM” list, created as a single reference for addressing genomic regions prone to mapping artifacts, highlights distinctions from both the GitHub Blacklist and the “Kundaje Unified” list.

To explore similarities between exclusion lists, we visualized Jaccard overlap indexes among them using multi-dimensional scaling (Methods). The “GitHub Blacklist” and our “Generated Blacklist” showed similarity, while the “Kundaje Unified,” “High Signal,” “Low Mappability,” and “HS + LM” lists appeared more distinct (Fig. 1D). To account for differences in list size, region number, and genome coverage, we applied the Forbes width overlap coefficient, known for its robustness in such contexts (Salvatore et al. 2020). This analysis indicated that the “Kundaje Unified” and “High Signal” lists share some similarities and align more closely with the “GitHub Blacklist,” whereas the “Low Mappability” and “HS + LM” lists diverge (Supplementary Results). These findings highlight notable heterogeneity among exclusion lists, suggesting that no single reference set universally defines problematic regions.

### 3.3. Excessive high signal annotation of blacklist-generated regions

Exclusion sets produced by the Blacklist software include annotations indicating whether regions are classified as “High Signal” or “Low Mappability,” with no overlap between categories. We observed that “High Signal” annotations were predominant across all organisms’ lists (83.75±9.54%), with the hg38 “GitHub Blacklist” showing a particularly strong bias (93.40%, Additional File 2). To investigate this pattern, we examined the Blacklist software’s algorithm and original C code (Supplementary Results). Our analysis revealed that the software defaulted to “High Signal” annotations in cases of ambiguity, as it could not output combined classifications. We addressed this by modifying the code to allow for a new annotation, “High Signal, Low Mappability,” and have made the updated version available (Code Availability).

The modified code produced consistent results (Additional File 1) and, for the hg38 genome assembly, identified the same 65 “Low Mappability” regions spanning 7.4 Mbp. Of the 1,208 “High Signal” regions (covering 263.8 Mbp), 183 were reclassified as “High Signal, Low Mappability.” Although these reclassified regions were few, they accounted for 219.4 Mbp, representing 80.88% of the total genome coverage in the Blacklist-generated hg38 exclusion set. Similar trends were observed for the mm10 assembly (Additional File 1). These findings underscore the importance of recognizing multi-annotation regions in exclusion set generation and highlight the potential for annotation biases to influence downstream analyses.

Recognizing the sensitivity of the Blacklist software to input settings, we systematically evaluated the effects of three parameters on the resulting exclusion sets. These parameters included the number of files (ranging from 10 to 300) to determine the optimal number needed for exclusion set generation, the “bridge” parameter (tested at 1000, 10 000, and the default 20 000) to assess the optimal merging distance for nearby exclusion regions, and the “k-mer” parameter (default 36, 50, and 100) to evaluate the ideal k-mer size. Notably, none of the parameter combinations produced exclusion sets closely resembling the GitHub Blacklist, the “Kundaje Unified” list, or our manually created High Signal and Low Mappability lists (Supplementary Results). These findings underscore the sensitivity of the Blacklist software to input data and internal parameters and highlight its limitations in replicating classical definitions of excludable regions, such as high-signal or low-mappability peaks.

### 3.4. Ribosomal genes are most affected by exclusion regions

To assess the impact of different exclusion sets on known transcripts, we quantified the extent to which these sets overlapped with transcripts (Additional File 5). Our analysis included two newer exclusion sets: the “Nordin CUT&RUN” list (Nordin et al. 2023) and the “GreyListChIP” list, generated using the GreyListChIP R package with a 1000 bp merging setting (Supplementary Results). We observed that the “GreyListChIP” list and the “High Signal” list affected the largest number of protein-coding genes, whereas the “Generated Blacklist” covered the greatest portion of gene bases (Fig. 2A). In contrast, the “Kundaje Unified” list had the smallest impact on the number and coverage of protein-coding genes, likely reflecting its manual curation, which intentionally avoids regions overlapping transcripts. Similarly, the “Nordin CUT&RUN” list showed a low impact on both the number and coverage of protein-coding genes. Comparable trends were observed for long noncoding RNAs and other transcript types (Fig. 2A).

We visualized a representative region containing ubiquitin-specific peptidase 17-like family member genes to illustrate the large size of the Blacklist-generated regions, the “Low Mappability” regions that harbor protein-coding genes, the tendency of “High Signal” regions to overlap genes, and the conservative approach of the “Kundaje Unified” and the “Nordin CUT&RUN” lists, which avoid overlapping genes (Fig. 2B, upper panel). These results indicate that the Blacklist-generated list, “High Signal,” “Low Mappability,” and the combined lists may remove peaks overlapping gene regions.

To determine whether specific gene sets or pathways are affected by exclusion regions, we performed KEGG enrichment analysis (Additional File 5). Pathways related to “Ribosome” and “Ribosome biogenesis in eukaryotes” were enriched in genes affected by the “Nordin CUT&RUN” list, the “High Signal” regions, the combined “HS + LM” regions, and our “Generated Blacklist” (Fig. 2C). Genes from these pathways were also affected by the “Low Mappability” and “GitHub Blacklist” regions, though the observed enrichments were not statistically significant. This result was expected, as ribosomal RNA (rRNA) constitutes approximately 80–90% of total cellular RNA (Kobayashi 2014, Warmerdam and Wolthuis 2019, Chen et al. 2020) and ribosomal RNA gene (rDNA) fragments are a well-characterized source of repeats (Kopylova et al. 2012). However, the “GitHub Blacklist,” “Kundaje Unified,” and “GreyListChIP” lists did not show any significant enrichment.

We visualized a cluster of ribosomal genes affected by the “Nordin CUT&RUN” list (Fig. 2B, lower panel), highlighting a key distinction between the “GitHub Blacklist” and the “Generated Blacklist.” The latter covered ribosomal genes, whereas the former did not, suggesting that the “GitHub Blacklist” may have been filtered to minimize overlap with gene regions. These findings underscore the unique characteristics of the “Nordin CUT&RUN” list, which, despite affecting fewer genes overall, appears to exhibit exclusive enrichment in ribosomal genes.

We examined the impact of exclusion sets on cancer-associated genes using oncoEnrichR analysis (Nakken et al. 2023). The analysis identified five oncogenes and tumor suppressors (collectively referred to as cancer drivers) with moderate to very strong evidence of being affected by the “GitHub Blacklist,” with KDM5A (lysine demethylase 5A) and MLH1 (mutL homolog 1) being the most notable oncogene and tumor suppressor, respectively (Additional File 6). The “Generated Blacklist” impacted 15 cancer drivers, including the NOTCH2 oncogene and tumor suppressor, with very strong confidence. The “High Signal” list affected 24 cancer drivers, encompassing key genes such as PIK3CA, MTOR, RAF1, and JUN. The “Low Mappability” list impacted six cancer drivers, including NOTCH2 and SSX2 (SSX family member 2). The combined “HS + LM” list affected the highest number of cancer drivers (32), while the “GreyListChIP” list impacted 17, including NOTCH2 and TP53. In contrast, the “Kundaje Unified” and “Nordin CUT&RUN” lists did not overlap with cancer driver genes. These observations suggest that the “High Signal” and “Low Mappability” regions should not be excluded and position the “Kundaje Unified” list as a viable option for analyses aiming to minimize the impact on functional pathways and oncogenes.

### 3.5. Using “sponge” sequences decreases artificial correlation in ChIP-seq data comparable to the blacklist-generated exclusion sets

**Figure 3:**
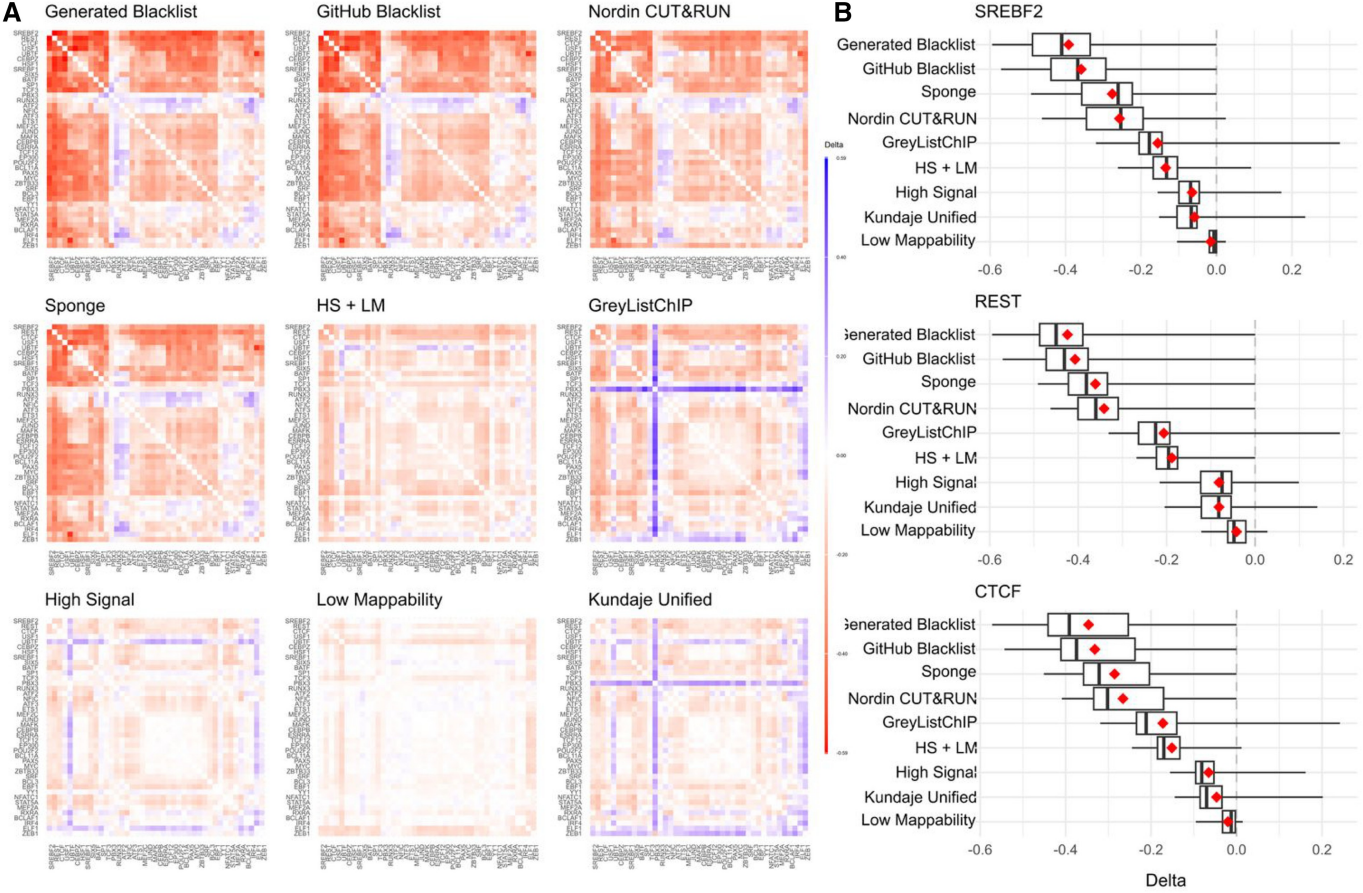
Changes in ChIP-seq signal correlation with and without reads overlapping exclusion sets or aligned to the sponge. Data for the Gm12878 cell line is shown. (A) Heatmaps of correlation differences for each exclusion set, sorted by mean (red/blue gradient corresponds to decreases/increases in correlations, respectively); (B) Correlation difference distributions for the top three most affected transcription factors.

The original Blacklist publication suggested that removing reads overlapping exclusion regions would enhance the biological interpretability of ChIP-seq data by reducing signal correlation among individual profiles. The authors hypothesized that read pileups in exclusion regions lead to spurious correlations and that removing them would allow for better biological interpretability. We evaluated this hypothesis by correlating ChIP-seq transcription factor signals for the GM12878 cell line before and after removing reads overlapping exclusion regions. Notably, removing reads overlapping the “High Signal,” “Low Mappability,” and “Kundaje Unified” lists resulted in the smallest decrease in correlations (Fig. 3A). We confirmed that removing reads overlapping the “Generated Blacklist” and the “GitHub Blacklist” lists resulted in the largest decrease in signal correlation for the REST, SREBF2, and CTCF transcription factors (Fig. 3B).

Aligning sequencing reads to a genome that includes so-called “sponge” sequences represents another strategy to reduce artifact signals. These sequences include satellite DNA, ribosomal DNA, mitochondrial sequences, etc., and constitute roughly 8% of the human genome typically omitted from the reference assembly (Miga et al. 2015). We hypothesized that including these “sponge” sequences at the alignment step would reduce read pileups and signal correlation compared to reads aligned to the assembled chromosomes only. We found that this was indeed the case, with the overall “sponge”-aligned signal correlation reduction being second only to the “Generated Blacklist” and “GitHub Blacklist” lists (Fig. 3). With minor exceptions, these observations were consistent for other transcription factors (Supplementary Results). In summary, these results position the “sponge” alignment as a viable alternative to fixed region-based read filtering.

### 3.6. “Sponge” sequences and the T2T-CHM13 alignment preserve biological signal in omics data

We hypothesized that using the “sponge” sequences would minimally affect or improve biological signals in omics data. We utilized two versions of the hg38 genome assembly: one that included only autosomal sequences, and another, referred to as “full plus sponge,” that additionally included unplaced contigs and the “sponge” sequences. To investigate whether the telomere-to-telomere human genome assembly could substitute for the effect of “sponge” sequences and contigs by having them properly assembled, we further utilized the T2T-CHM13 genome assembly (Nurk et al. 2022).

We analyzed RNA-seq data from patient-derived xenograft (PDX) models of breast cancer and found that the total number of reads overlapping gene regions remained nearly unchanged (*P*-value > .3, pairwise Wilcoxon test). Principal Component Analysis (PCA) also demonstrated that the alignment strategy preserved biological signal and did not introduce batch effect-like variability (Supplementary Results). We performed differential gene expression analysis and found that inclusion of contigs and “sponge” sequences in the hg38 genome assembly resulted in significant downregulation of six genes (three noncoding and three ribosomal). Comparison of data aligned to the T2T-CHM13 assembly versus the hg38 assemblies resulted in 1392/1377 upregulated and 495/503 downregulated genes for hg38 autosomes vs. T2T and hg38 full plus sponge vs. T2T, respectively. These gene sets were highly overlapping, and the majority of them (56%) were ncRNAs, while only 34% were protein-coding (Additional File 8). Notably, approximately 3% of those genes were in regions specific to the T2T-CHM13 assembly, indicating that the majority of the detected genes are likely due to their improved assembly. To understand their functional significance, we performed KEGG pathway enrichment analysis. We found clusters of histone genes associated with the immune system upregulated in the T2T-CHM13–aligned data (Additional File 8). This is expected, as histone gene regions are repetitive, contain multiple highly similar copies, and were difficult to resolve in the hg38 genome assembly (Nurk et al. 2022). Among downregulated genes, we observed members of the Speedy/RINGO cell cycle regulator family, which encode cyclin-like proteins that bind and activate cyclin-dependent kinases (CDKs) and play roles in cell cycle and growth (Additional File 8). This is also expected, as the SPDYE gene family consists of multiple tandemly arrayed paralogs resolved in the T2T genome assembly, making them a high-copy, highly similar cluster that was hard to resolve in hg38 (Vollger et al. 2022). These results highlight the potential for T2T-CHM13 alignment to provide richer biological insights.

ATAC-seq represents another data type that may benefit from alignment with “sponge” sequences. We compared the number of peaks differentially accessible between treated and untreated conditions (see Methods) and found that including alternative contigs and “sponge” sequences reduced the number of peaks by approximately 3.7% (49 496/47 583 peaks in hg38 autosomal/full plus sponge alignment settings). Alignment to the T2T-CHM13 genome assembly resulted in the largest number of peaks (49 538; Additional File 9). This is expected, as the T2T-CHM13 genome assembly should include previously unplaced contigs and/or sponge sequences. Peaks detected under each alignment strategy had similar widths (median 248 bp, *t*-test *P*-value > .3). We annotated peaks with genes and found the majority overlapped between the hg38 autosomal and full plus sponge alignments (Additional File 9). Alignment to the T2T-CHM13 genome assembly resulted in the largest number of unique genes (6360), 305 of which were protein-coding. The hg38 autosomal-only alignment resulted in peaks associated with 195 unique genes (53 protein-coding). By contrast, the hg38 full plus sponge alignment resulted in 54 unique genes (28 protein-coding; Supplementary Results). To assess the functional importance of these unique genes, we performed KEGG pathway enrichment analysis. No significant enrichments were found in any of the unique gene sets, suggesting that different alignment strategies do not affect specific functional categories. However, closer inspection of the unique protein-coding genes in the hg38 full plus sponge alignment revealed several clinically relevant genes, such as FOS (oncogene, AP-1 complex), BIK (BCL2-Interacting Killer, tumor suppressor, apoptosis regulator), FANCE (Fanconi anemia complementation group E), and ECT2 (Epithelial Cell Transforming 2, oncogene). Similarly, unique genes detected under the T2T alignment included several well-studied cancer-related genes, including FOXA1 (key transcription factor in hormone-responsive cancers), BTG2 (tumor suppressor, p53 target, regulator of cell cycle arrest), FOSL2 (AP-1 transcription factor family, proliferation and cancer progression), MAF (oncogenic transcription factor in multiple myeloma), and E2F2 (cell cycle regulator). Similar to RNA-seq, these results suggest that the T2T-CHM13 alignment strategy may improve biological signal, and that alignment with “sponge” sequences may also enhance biologically relevant signal.

We further investigated the impact of “sponge” sequences on single nucleotide polymorphism (SNP) and InDel calling from whole-genome sequencing (WGS) data. When benchmarking against established databases (dbSNP) and high-confidence truth sets (GIAB), we found that data aligned to the hg38 assembly with sponge sequences exhibited the highest proportion of previously annotated variants (Supplementary Results). In contrast, the standard hg38 autosomal alignment yielded a higher frequency of variants lacking database support. While the T2T-CHM13 alignment expectedly produced a substantial number of unannotated variants due to its inclusion of novel genomic regions, the majority of calls unique to either the T2T or standard hg38 autosomal assemblies did not overlap with GIAB benchmarks (Supplementary Results). Collectively, these results suggest that incorporating sponge sequences into the hg38 reference minimizes the detection of unannotated variants and improves the recovery of well-characterized SNPs and InDels.

We also ran the Blacklist software on reads realigned to the hg38 full plus sponge and the T2T-CHM13 genome assemblies. As compared with the hg38 autosome-aligned reads, the Blacklist software identified more regions (4029 hg38 full sponge and 3192 T2T-CHM13 vs. 1273 hg38 autosome), and they were narrower (75 810 bp and 13 292 bp vs. 213 093 bp). This is expected as the sponge sequences and the full T2T assembly would diminish pileups formed by improperly aligned reads causing the Blacklist software to focus on smaller but more abundant peaks. Considering reproducibility and parameter uncertainty with the Blacklist algorithm, we recommend the hg38 full plus sponge or the T2T-CHM13 genome assemblies to diminish the effect of artefact signals at the alignment step.

## 4. Discussion

The original publication describing the Blacklist software provides only limited methodological details, which hinders the reproducibility of its results. For instance, key algorithmic parameters such as k-mer size, “binSize,” “binOverlap,” and the single- versus multi-read configuration of Umap mappability files are not described or benchmarked. Additionally, the correlation analysis demonstrating reduced artificial correlations among ChIP-seq data lacks transparency regarding data sources and correlation methods, prompting us to implement our own version of this analysis. Moreover, the data properties influencing exclusion set generation remain unexplored, leaving room for variability across different datasets and software configurations. In this study, we addressed these gaps by reverse-engineering the Blacklist software, fixing an annotation error, providing corrected C++ and Python code, and systematically investigating the properties of various exclusion sets and algorithmic choices. While our Blacklist implementation produced identical results to the original code, they differed from the exclusion sets provided in the Blacklist GitHub repository. Given the poor reproducibility of the original Blacklist software and its sensitivity to internal parameters and input data, we advise against relying solely on static exclusion lists and instead recommend adopting upstream alignment strategies—such as “sponge” sequences or T2T-CHM13—to more effectively mitigate artifact signals.

While the hg38 genome assembly remains integral to current genomics research, we recommend utilizing “sponge” sequences during alignment as a robust alternative to applying exclusion sets. We demonstrate that including “sponge” sequences reduces artifact signals comparable to Blacklist-generated lists. The fact that some reads overlapping excludable regions remain aligned even in the presence of “sponge” sequences suggests that removing all reads from excludable regions may lead to loss of biologically relevant signal. “Sponge” sequences avoid the problem of poor reproducibility of exclusion sets and potential loss of biological signal by targeting artifact reads at the alignment step. We observed their effectiveness in RNA-seq alignment (negligible effect on gene expression), ATAC-seq (improving biological signal around clinically relevant genes), and in whole genome sequencing settings (minimal loss of genomic variants that are likely false positives), suggesting that including “sponge” sequences as part of genome references may improve biological signal from genomic data generated by any short-read technology.

Although highly promising, using “sponge” sequences remains a method that is less broadly applicable. This is due to a limited understanding of the sequences that constitute “sponge” or “decoy” libraries. While these sequences have been assembled for the hg38 human genome (Miga et al. 2015), they have not yet been created for genome assemblies of other model organisms. Moreover, with the development of telomere-to-telomere human (Nurk et al. 2022) and mouse (Liu et al. 2024) genome assemblies these “sponge” sequences may not be necessary as demonstrated by a recent benchmark of the T2T-CHM13 genome assembly on genomic variants (Cherchi et al. 2025). The development of long-read sequencing (Oxford Nanopore Technologies, Pacific Biosciences) may also alleviate spurious alignments, as we have shown when comparing the 36 bp and 101 bp alignments (Supplementary Results). Furthermore, the growing amount of whole genome data and the development of pangenome graph assembly methods has been shown to improve genomic variant discovery, RNA-seq and chromatin immunoprecipitation read mapping (Liao et al. 2023). Pangenome graph assembly represents a promising way to alleviate alignment artifacts; however, these methods are still relatively less widely adopted than those using linear genome assemblies. Our future work includes defining “sponge” sequences for other genomes and model organisms as well as exploring the use of the pangenome graph assembly to improve biological signal and eliminate the need for exclusion sets.

## Supplementary Material

btag110_Supplementary_Data

## Data Availability

The modified Blacklist code and scripts to reproduce our analyses and figures are available at https://github.com/dozmorovlab/excluderanges_supplementary and at https://doi.org/10.5281/zenodo.18090597. The GreyListChIP data generated in this work is available as a part of the excluderanges R package.
