# Supplementary material for: Beyond blacklists: a critical assessment of exclusion set generation strategies and alternative approaches
Source: Bioinformatics. 2026 Mar 13;42(3):btag110. doi: 10.1093/bioinformatics/btag110 (PMC13020910; doi:10.1093/bioinformatics/btag110)
Supplement: btag110_Supplementary_Data [file btag110_supplementary_data.zip › Additional_files_legend.docx]

# Additional file legends

## Additional File 1

**Supplementary Table S1. Dominant High Signal annotation counts and proportions in exclusion sets generated by the Blacklist software.** The “Corrected List” refers to exclusion sets generated using our modified version of the Blacklist software, which enables dual high-signal and low-mappability annotations.

## Additional File 2

**Supplementary Table S2. Diverse characteristics of human sequencing data used by the Blacklist software.** “bam_accession_table” – Accession numbers of 1,255 BAM files and the associated FASTQ files (“R1 FASTQs”, “R2 FASTQs”), their paired-end status, and indicators of whether the data were cropped, pooled, duplicated, or restricted. Also includes BAM read lengths and the number of mapped and unmapped reads. “donor_accession_table” – Accession numbers of 250 donors and the associated BAM files, total mapped and unmapped reads per donor, the number of BAM files per donor, read lengths, and tissue ontology. “fastq_accession_table” – Characteristics of the original FASTQ files used to generate sample-specific BAM files.

## Additional File 3

**Supplementary Table S3. Characteristics of exclusion sets *generated using* different aligners.** Total number and average width of the regions, number of regions overlapping gaps, and proportion of gap coverage.

## Additional File 4

**Supplementary Table S4. Characteristics of exclusion sets generated with different parameters.** Total number and average width of the regions, number of regions overlapping gaps, and proportion of gap coverage. The list names include the bridge parameter (e.g., b1000), the k-mer parameter (e.g., k36), and the number of files utilized (e.g., n50).

## Additional File 5

**Supplementary Table S5. Genes affected by exclusion sets.** Protein-coding genes, sorted alphabetically, are listed first, followed by lncRNAs and other gene categories.

## Additional File 6

**Supplementary Table S6. Oncogenes and tumor suppressors affected by exclusion sets.** “Cancer Association” results from the oncoEnrichR analysis are shown for each list, sorted by oncogenic and tumor suppressor confidence levels.

## Additional File 7

**Supplementary Table S7. Alignment statistics of ChIP-seq input sequencing data to the “sponge” sequences and other contigs comprising the human genome reference.**

## Additional File 8

**Supplementary Table S8. Differentially expressed genes *identified* between samples aligned to the hg38 autosomal reference, the hg38 reference with the “sponge” sequences (hg38_full_sponge), and the T2T-CHM13 genome assembly.** Sheets are named by the comparisons performed. The file includes KEGG pathway enrichment analyses for up- and downregulated genes.

## Additional File 9

**Supplementary Table S9. Annotated ATAC-seq peaks detected from data aligned to the hg38 autosomal reference, the hg38 reference with the “sponge” sequences (hg38_full_sponge), and the T2T-CHM13 genome assembly.** Peaks were detected relative to the corresponding control and annotated with the nearest genes.
